# No Evidence of Metabolomic Disruptions From Real‐World Intakes of Aspartame or Saccharin: The Coronary Artery Risk Development in Young Adults Study

**DOI:** 10.1111/1753-0407.70138

**Published:** 2025-08-12

**Authors:** Brian T. Steffen, Elizabeth R. Lusczek, David R. Jacobs, Chi Chen, Venkatesh L. Murthy, Linda Van Horn, James G. Terry, John Jeffrey Carr, Lyn M. Steffen

**Affiliations:** ^1^ Division of Computational Health Sciences, Department of Surgery University of Minnesota School of Medicine Minneapolis Minnesota USA; ^2^ Division of Epidemiology and Community Health School of Public Health, University of Minnesota Minneapolis Minnesota USA; ^3^ Department of Food Science and Nutrition University of Minnesota St. Paul Minnesota USA; ^4^ Departments of Medicine and Radiology University of Michigan Ann Arbor Michigan USA; ^5^ Nutrition Division, Department of Preventive Medicine, Feinberg School of Medicine Northwestern University in Chicago Chicago Illinois USA; ^6^ Department of Radiology and Vanderbilt Translational and Clinical Cardiovascular Research Center (VTRACC) Vanderbilt University Medical Center Nashville Tennessee USA

**Keywords:** artificial sweeteners, aspartame, diet, metabolomics, saccharin

## Abstract

**Background:**

Artificial sweeteners have become ubiquitous additives in the food supply, and yet the safety of their regular consumption remains controversial. The present study examined whether intakes of aspartame or saccharin are related to aberrations in the plasma metabolome indicating disruptions in metabolism.

**Methods:**

A cohort of 2160 male and female participants, mean age 32.1 years, was included in the analysis. Liquid chromatography and mass‐spectrometry assessed 549 unique plasma metabolites. Diet was assessed using a validated questionnaire that allowed for estimation of aspartame and saccharin intakes. A generalized linear regression model evaluated associations of saccharin or aspartame intake with plasma metabolites with adjustment for potential confounders and multiple comparisons. Multiple sensitivity analyses and propensity score matching were conducted.

**Results:**

Heavy aspartame intake (≥ 5 servings/day) was associated with plasma levels (per SD) of saccharin (*β* = 0.90; *q* = 9.0E‐36), myo‐inositol (*β* = 0.27; *q* = 3.7E‐04), caffeine (*β* = 0.31; *q* = 4.1E‐04), and five metabolites of caffeine including 1,7‐dimethyluric acid (*β* = 0.37; *q* = 7.1E‐06), 1‐methylurate (*β* = 0.36; *q* = 7.1E‐06), 5‐acetylamino‐6‐amino‐3‐methyluracil (*β* = 0.38; *q* = 3.2E‐6), theophylline (*β* = 0.36; *q* = 9.1E‐06), and 1‐methylxanthine (*β* = 0.32; *q* = 2.0E‐03). Saccharin intake was associated with plasma levels of saccharin alone (*β* = 0.29; *q* = 1.8E‐10). No associations with sugars, carbohydrates, lipids, amino acids, or other metabolites that would suggest metabolic perturbations were observed with either artificial sweetener; sensitivity analyses supported these findings.

**Conclusions:**

In the largest metabolomics study to date, no link was found between metabolic disruptions and either aspartame or saccharin intake. We cannot exclude the possibility that more extreme intakes may be related to metabolic disruptions among consumers of artificial sweeteners.

## Graphical abstract

Consumption of aspartame or saccharin was not significantly related to plasma apart from caffeine metabolites. These findings do not support a link between real‐world intakes of aspartame or saccharin and metabolic disruptions; this has implications for treating physicians and patients concerned about artificial sweeteners.

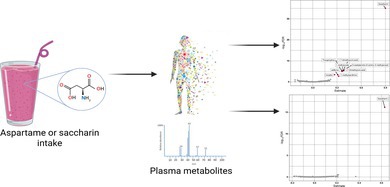

**Summary**

- Consumption of aspartame or saccharin was not significantly related to over 540 plasma metabolites, consistent with a null effect of these artificial sweeteners on metabolism.
- The rigor of these findings was confirmed by multiple sensitivity analyses, and propensity score matching.
- These findings do not support a link between real‐world intakes of aspartame or saccharin and metabolic disruptions, which have implications for treating physicians and patients worried about artificial sweeteners.

## 1 Introduction

Aspartame and saccharin are two of the most widely used artificial sweeteners (ArtSws) in the US [1] and are found in “zero sugar”, “diet”, and “lite” branded foods and beverages including sodas, fruit juices, breads, cereals, and yogurts. ArtSws have been FDA approved as safe non‐caloric sugar substitutes based on short‐term interventions and trials [2, 3]. And yet, these studies may not be ideal for detecting the potential effects of daily, real‐world ArtSw intakes in a free‐living cohort. Indeed, regular consumption of ArtSws has been associated with metabolic dysfunction and greater adiposity [4, 5, 6, 7, 8].

In one of the largest prospective studies of individual ArtSw intake, we showed that habitual intake of aspartame or saccharin was associated with greater visceral and intermuscular adipose tissue volumes as well as risk of developing obesity over a 17.5 year follow‐up independent of caloric intake and diet quality in over 2000 participants of the Coronary Artery Risk Development in Young Adults (CARDIA) study [4], which was consistent with other observational studies [2, 3, 5, 8]. In addition, experimental evidence has shown that ArtSws disrupt glucose homeostasis [7], lipid metabolism [9], lipid peroxidation [10], and adiponectin and leptin regulation [11]. However, whether these experimental findings translate to humans remains controversial and requires further investigation.

Using a metabolomics‐based approach, the present study tested whether intakes of distinct ArtSws are related to plasma metabolites including amino acids, carbohydrates, and lipids that may indicate metabolic disruptions. Critically, this study was designed to address the primary limitation of previous studies of ArtSw—bias due to reverse causation. Specifically, it has been proposed that individuals with declining metabolic health or who are otherwise gaining weight may begin consuming more “diet” and “lite” branded products to reduce caloric intake, thereby biasing the association between ArtSw intake and subsequent metabolic outcomes. A primary rationale for this study was to apply multiple approaches to control for this scenario in testing whether aspartame or saccharin consumers show any metabolic perturbations independent of this bias.

## 2 Methods

### 2.1 Study Population

CARDIA is a prospective cohort study of young adults with the objective of identifying risk factors for coronary heart disease development [12]. At baseline, the cohort was composed of 5115 Black and White women and men, aged 18–30 years (1985–1986), who were recruited across four U.S. metropolitan areas: Birmingham, AL; Chicago, IL; Minneapolis, MN; and Oakland, CA. For this analysis, Year 7 CARDIA Exam data were used, and 3943 participants attended this exam visit. Institutional Review Boards at each field center approved CARDIA study protocols. All participants gave written informed consent for participation at each CARDIA exam.

### 2.2 Data Collection

Demographic characteristics (age, sex, race, and education) and lifestyle factors (physical activity, cigarette smoking, and alcohol intake) were self‐reported at Year 7 using questionnaires. A separate questionnaire was administered to assess physical activity [13] at Year 7, which was scored based on time spent in activities and weighted by estimated energy expenditures. Stadiometers and beam balance scales were used to measure height and weight, respectively. BMI was calculated as weight in kilograms divided by height in meters squared (kg/m^2^).

### 2.3 Diet Assessment

Trained and certified interviewers administered the CARDIA Diet History, a validated questionnaire used to assess usual dietary intake of the previous month [14] at exam Year 7. The dietary data entry and analysis software, Nutrition Data System for Research (NDSR), was developed at the University of Minnesota Nutrition Coordinating Center and used to code dietary data into food and beverage groups and nutrient composition, including the ArtSw (mg/d) aspartame and saccharin exposure variables. To evaluate diet quality, the Healthy Eating Index 2020 (HEI2020), which assesses compliance with the 2015–20 Dietary Guidelines for Americans, was derived from food and beverage group (servings/day) data consisting of fruit, fruit juice, vegetables, legumes, nuts, whole grain products, refined grain products, dairy, eggs, meat, fish and seafood, candy, coffee, tea, sugar‐sweetened beverages, and diet beverages [15]. Participants were asked to provide details about foods and beverages, including brand name information, which allowed estimation of artificial sweetener intakes.

### 2.4 Plasma Collection and Metabolomics Assessment

For the CARDIA cohort, standard procedures for blood collection, processing, storage, and quality control have been described previously [16]. Briefly, at the Year 7 exam visit, blood was collected from fasting participants by trained technicians with phlebotomy experience. No more than 90 min was permitted between the blood draw and placement in a −70°C freezer. Metabolomics assessment was conducted at the Broad Institute of Harvard‐MIT (Cambridge, MA), and details of the liquid chromatography and mass‐spectrometry platforms have been previously described [17, 18]. The Broad Institute of Harvard‐MIT (Cambridge, MA) conducted the metabolite profiling of lipids (“C8‐positive”), amino acids and related polar metabolites and sugars (“HILIC‐positive” and “HILIC‐negative”), and free fatty acids, lipid‐derived mediators and bile acids (“C18‐negative”). In total, 608 metabolomic analytes were available for analysis and represented 549 unique metabolites, which served as the outcome variables.

### 2.5 Aspartame and Saccharin Intakes

At the time of the Year 7 exam, only aspartame, saccharin, and acesulfame potassium were FDA approved for human consumption. Acesulfame potassium was not available for analysis but had only recently been approved by the FDA in 1988 (for use in beverages). It was not approved as a general‐purpose sweetener until 2003. Other sweeteners such as sucralose, erythritol, and stevia were not approved for human consumption at the time of the Year 7 exam.

The distribution of aspartame and saccharin intakes was highly skewed. For aspartame, 53% of the available cohort (*n* = 1154) reported no intake, and it was therefore categorized into zero intake, moderate intake (greater than zero mg and less than 175 mg or 5 servings per day), and heavy intake (≥ 5 servings per day). For saccharin, 90% of the cohort at Year 7 showed zero intake of saccharin, and its intake was therefore dichotomized into users and non‐users.

### 2.6 Statistical Analysis

Cross‐sectional analyses were conducted using SAS version 9.4 (SAS Institute Inc., Cary, NC) on Year 7 exam data. A total of 3943 CARDIA participants attended the Year 7 exam visit. Exclusion criteria included missing metabolomic data (*n* = 1638), missing demographic or lifestyle data, or implausible energy intake [< 600 and > 6000 kcal/d for women and < 800 and > 8000 kcal/day for men] (*n* = 145). A total of 2160 participants had the requisite diet, metabolomics, and covariate data. Participant characteristics are presented as means (±SD) for continuous variables and frequencies (%) for categorical variables across aspartame intake categories. Statistical significance was assessed using analysis of variance for continuous variables and chi‐square tests for categorical variables. Characteristics of users and non‐users of saccharin are likewise presented, and statistical significance was assessed using *t*‐tests for continuous variables and chi‐square tests for categorical variables.

A generalized linear model assessed relationships between the above categories of aspartame or saccharin intake and plasma metabolite outcomes with adjustments for age, sex, race, field center, education, body mass index, smoking status, self‐reported physical activity, total caloric intake, HEI2020 score, prevalent diabetes, use of blood pressure medications, and kidney function as estimated by plasma creatinine levels. Metabolite values were log‐transformed to normalize the data and were then standardized (mean = 0 and SD = 1). Metabolite values outside of 6 SD from the mean were excluded for that individual metabolite analysis. Results that met a Benjamini‐Hochberg false discovery rate (FDR ≤ 0.05) were considered statistically significant, and this accounted for 608 metabolite outcomes and included duplicated analytes across the metabolomic platforms.

A number of analyses were conducted to ensure the robustness of the main findings. First, propensity score matching (PSM) was performed. Propensity score matching aims to balance covariates between individuals with heavy and no aspartame or saccharin intake by matching them on their predicted probability (propensity score) of consumption of the artificial sweetener, conditional on measured covariates. For each ArtSw, propensity scores were generated using logistic regression of heavy aspartame or saccharin intake against age, sex, race, field center, education, smoking status, BMI, HEI2020 score, total caloric intake, and prevalent diabetes. Log‐odds coefficients provided point estimates to calculate propensity scores. For heavy aspartame and saccharin intake, consumers were matched 1:1 to non‐consumers based on propensity scores using a greedy matching algorithm with a caliper of 0.15 standard deviations of the logit of the propensity score. An exact match was enforced on race due to its strong association with intake of either ArtSw. Group balance was assessed using standardized mean differences, with < 0.1 indicating acceptable balance. All variables for matched groups showed standardized mean differences below this threshold, with most showing < 0.05 standardized mean differences. Using these matched cohorts, associations of each ArtSw exposure with plasma metabolites were estimated using a generalized linear regression model with the same transformed and standardized metabolite outcomes described in the primary analysis. Given the smaller sample sizes of the PSM analyses, adjustments were made for age, sex, BMI, and education to control for any residual confounding from these variables.

Two sensitivity analyses were performed. In the first analysis, we aimed to increase statistical power by examining aspartame and saccharin intakes as standardized continuous variables (per SD) following the removal of non‐consumers of the ArtSw. In the second analysis, we further tested whether relationships were independent of excess adiposity and removed possible confounding by restricting the sample to participants with a non‐overweight and non‐obese weight status (BMI < 25 kg/m^2^). Both analyses used the same generalized linear model described in the primary analysis. Other covariates including participant intakes of protein, fat, and carbohydrates were tested in sensitivity analyses but did not materially change the results.

## 3 Results

A total of 3943 CARDIA participants attended the Year 7 exam visit, all of whom had dietary intake data. Individuals with missing metabolomic data (*n* = 1638), missing demographic or lifestyle data, or implausible energy intake (< 600 and > 6000 kcal/d for women and < 800 and > 8000 kcal/day for men) (*n* = 145) were excluded.

Characteristics of 2160 CARDIA participants are shown for heavy, moderate, and non‐consumers of aspartame in Table 1. Moderate and heavy consumers averaged 25.4 and 295.9 mg of aspartame per day, respectively (1 serving = 35 mg). Individuals with higher intakes of aspartame were more likely to be White, female, and show greater mean levels of education, physical activity, and BMI. Mean total caloric intakes were lower among moderate (−324 kcal/day) and heavy aspartame consumers (−259 kcal/day) compared to non‐consumers, and non‐consumers showed the lowest mean HEI2020 score. Characteristics of 2160 CARDIA participants by saccharin intake are shown in Table 2. Mean saccharin intake among users was 10.6 mg/day. Similar to aspartame, saccharin users were more likely to be White, female, and show greater mean levels of education and BMI. Compared to non‐users, saccharin users showed significantly lower mean total caloric intakes (−273 kcal/day; *p* = 0.002) and modestly higher HEI2020 scores (+2.1; *p* = 0.007).

**TABLE 1:** Demographic, lifestyle, and anthropometric characteristics of CARDIA participants stratified by heavy, moderate, and non‐consumption of aspartame.

Factor	Dietary aspartame intake			
	Non‐user (*n* = 1148)	Moderate user (*n* = 751)	Heavy user (*n* = 261)	*p*
Aspartame (mg/day), median (IQR)	0.0 (0.0, 0.0)	25 (9, 71)	296 (196, 412)	
Age, mean (SD)	31.9 (3.7)	32.3 (3.5)	32.9 (3.3)	< 0.001
Female, *n* (%)	456 (39.7)	381 (50.7)	140 (53.4)	< 0.001
Black, *n* (%)	522 (45.5)	279 (37.2)	33 (12.6)	< 0.001
Education, *n* (%)				
1–12 Years	363 (31.6)	137 (18.2)	37 (14.1)	< 0.001
13–16 Years	635 (55.3)	399 (53.1)	134 (51.1)	
> 16 Years	150 (13.1)	215 (28.6)	91 (34.7)	
Field center, *n* (%)				
Birmingham, AL	255 (22.2)	140 (18.6)	53 (20.2)	< 0.001
Chicago, IL	254 (22.1)	158 (21.0)	82 (31.4)	
Minneapolis, MN	311 (27.1)	176 (23.4)	75 (28.7)	
Oakland, CA	328 (28.6)	277 (36.9)	51 (19.5)	
Smoking status, *n* (%)				
Never	659 (57.4)	509 (67.8)	149 (57.1)	< 0.001
Former	165 (14.4)	127 (16.9)	63 (24.1)	
Current	324 (28.2)	115 (15.3)	49 (18.8)	
Physical activity (EUs), median (IQR)	293 (152, 515)	319 (181, 552)	316 (150, 521)	0.07
Total intake (kcal), median (IQR)	2697 (2023, 3747)	2373 (1898, 3201)	2438 (1842, 3043)	< 0.001
HEI2020 score, mean (SD)	54.6 (10.8)	58.1 (10.0)	56.7 (10.3)	< 0.001
BMI (kg/m^2^), mean (SD)	25.3 (4.6)	25.9 (5.3)	26.6 (5.0)	< 0.001
Prevalent diabetes, *n* (%)	14 (1.2)	18 (2.4)	6 (2.3)	0.19

**TABLE 2:** Demographic, lifestyle, and anthropometric characteristics of CARDIA participants stratified by saccharin intake.

Factor	Dietary saccharin intake		*p*
	Non‐users (*n* = 1941)	Users (*n* = 219)	
Saccharin (mg/day), median (IQR)	0.0 (0.0, 0.0)	10.6 (4.0, 29.5)	
Age, mean (SD)	32.2 (3.6)	32.0 (3.5)	0.65
Female, *n* (%)	842 (43.4)	134 (61.2)	< 0.001
Black, *n* (%)	862 (44.4)	76 (34.7)	0.006
Education, *n* (%)			
1–12 Years	502 (%)	35 (%)	0.005
13–16 Years	1037 (%)	130 (%)	
> 16 Years	402 (%)	54 (%)	
Field center, *n* (%)			
Birmingham, AL	387 (20)	61 (28)	< 0.001
Chicago, IL	459 (24)	35 (16)	
Minneapolis, MN	489 (25)	73 (33)	
Oakland, CA	606 (31)	50 (23)	
Smoking status, *n* (%)			
Never	1183 (61)	134 (61)	0.10
Former	310 (16)	45 (21)	
Current	448 (23)	40 (18)	
Physical activity (EUs), median (IQR)	305 (158–530)	316 (170–508)	0.85
Total caloric intake, median (IQR)	2856 (1261)	2584 (1152)	0.002
HEI2020 score, mean (SD)	55.8 (10.6)	57.9 (10.1)	0.007
Prevalent diabetes, *n* (%)	30 (2)	10 (5)	0.002
BMI (kg/m^2^), mean (SD)	25.6 (4.8)	26.3 (5.7)	0.03

**FIGURE 1:**
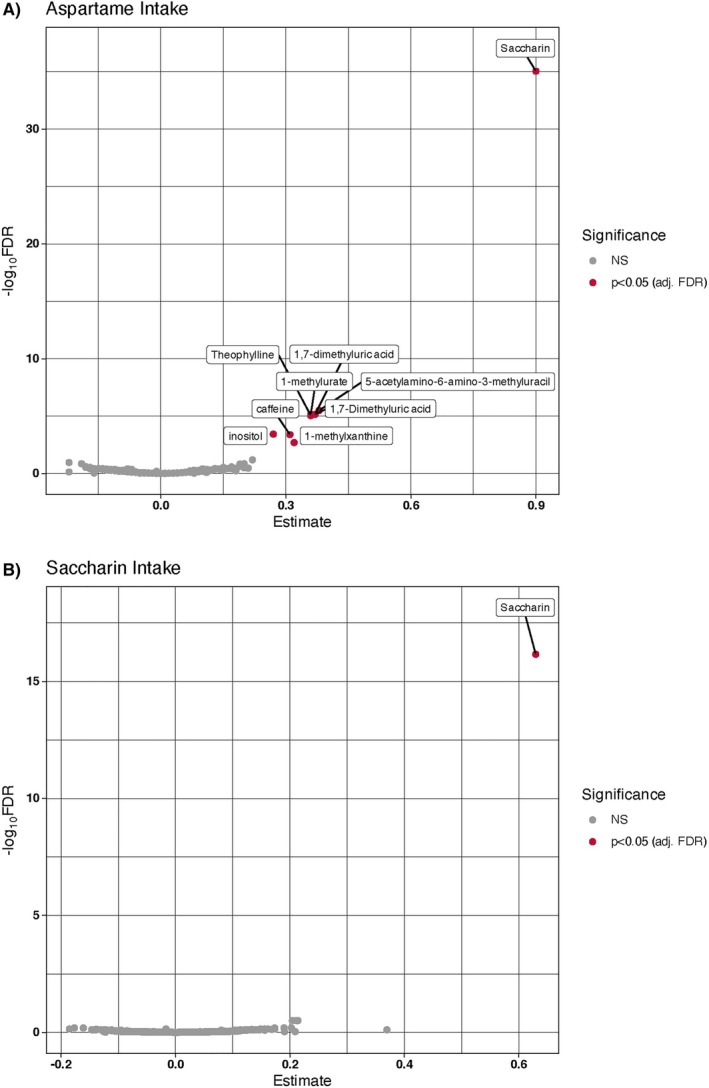
Volcano plots of plasma metabolites associated with intakes of (A) heavy aspartame (≥ 5 servings/day); and (B) saccharin (> 0 servings/day).

In Figure 1, associations of plasma metabolites (per SD) with intakes of (A) heavy aspartame (≥ 5 servings/day); and (B) saccharin (> 0 servings/day) are shown. Heavy aspartame intake was associated with plasma levels of saccharin (*β* = 0.90; *q* = 9.0E‐36), myo‐inositol (*β* = 0.27; *q* = 3.7E‐04), caffeine (*β* = 0.31; *q* = 4.1E‐04), and five metabolites of caffeine including 1,7‐dimethyluric acid (*β* = 0.37; *q* = 7.1E‐06), 1‐methylurate (*β* = 0.36; *q* = 7.1E‐06), 5‐acetylamino‐6‐amino‐3‐methyluracil (*β* = 0.38; *q* = 3.2E‐6), theophylline (*β* = 0.36; *q* = 9.1E‐06), and 1‐methylxanthine (*β* = 0.32; *q* = 2.0E‐03). Moderate aspartame intake (< 5 servings) was associated with plasma saccharin alone (*β* = 0.26; *q* = 4.4E‐6). Shown in Figure 1B, saccharin intake (> 0 servings/day) was associated with plasma levels of saccharin (*β* = 0.29; *q* = 1.8E‐10).

**FIGURE 2:**
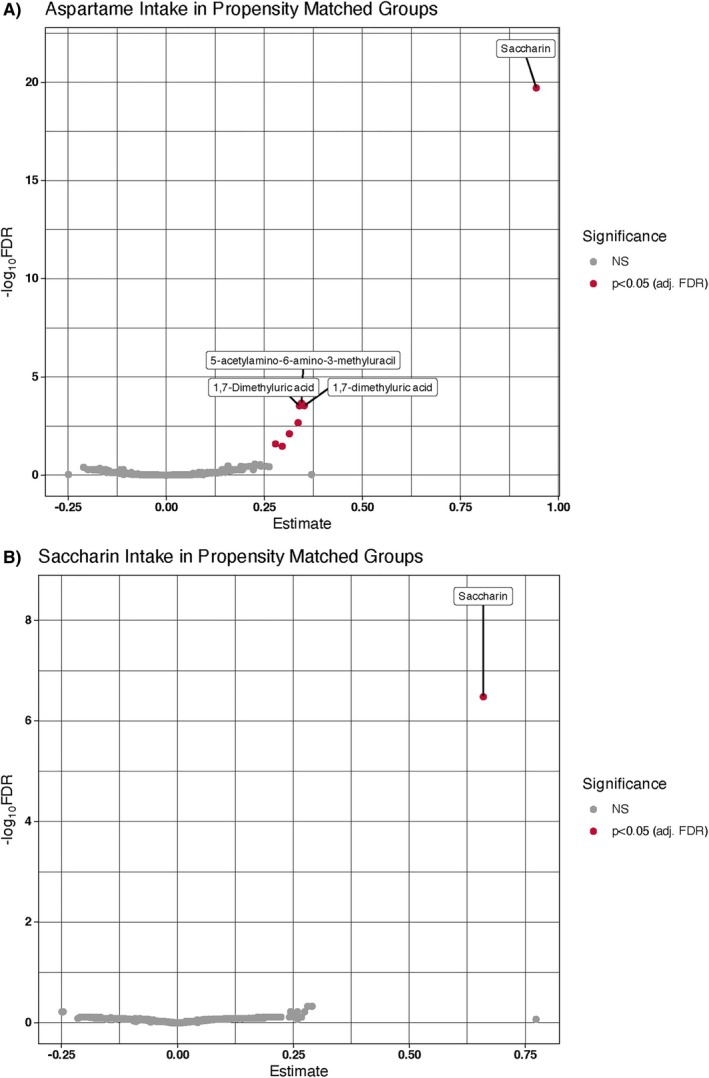
Results of propensity score matching analysis. Volcano plots of plasma metabolites associated with intakes of (A) heavy aspartame (≥ 5 servings/day); and (B) saccharin (> 0 servings/day).

To evaluate the robustness of these findings, PSM analyses were performed. Results are shown in Figure 2 and were largely consistent with the above main analysis. Heavy aspartame consumers (Figure 2A) showed significantly higher plasma levels (per SD) of saccharin (*β* = 0.94; *q* = 1.9E‐20), 5‐acetylamino‐6‐amino‐3‐methyluracil (*β* = 0.35; *q* = 2.2E‐04), two signals for 1,7‐dimethyluric acid (*β* = 0.35; *q* = 2.9E‐04), theophylline (*β* = 0.34; *q* = 2.1E‐03), 1‐methylurate (*β* = 0.31; *q* = 7.9E‐03), and caffeine (*β* = 0.29; *q* = 2.6E‐02). A signal for plasma glucuronate also reached significance (*β* = 0.30; *q* = 3.4E‐02). For saccharin PSM results, users showed significantly higher levels of plasma saccharin alone (*β* = 0.66; *q* = 3.1E‐07).

Additional sensitivity analyses were performed by restricting tests to ArtSw consumers. Among consumers, aspartame intake (*N* = 1012) was examined as a continuous variable (per SD), and associations are shown in Table 3. Results were similar to those identified in the main analysis, with one exception: an inverse association was detected for C40:6 phosphatidylcholine (*β* = −0.34; *q* = 3.1E‐03). An examination of saccharin intake as a continuous variable among consumers (*N* = 219) showed no associations that met the significance threshold, and all *q*‐values ≤ 0.8.

**TABLE 3:** Associations of plasma metabolites (SD) per 1 SD increase in aspartame intake among CARDIA participants, restricted to aspartame consumers (*N* = 1012).

Metabolite	Estimate	LowerCL	UpperCL	FDR	*n* ^a^
5‐acetylamino‐6‐amino‐3‐methyluracil	0.55	0.39	0.70	2.1E‐09	996
Saccharin	0.42	0.30	0.54	2.2E‐09	1007
1,7‐Dimethyluric acid	0.36	0.24	0.47	4.4E‐07	1001
1,7‐dimethyluric acid	0.23	0.15	0.30	4.4E‐07	998
1‐methylurate	0.45	0.29	0.61	5.4E‐06	994
1‐methylxanthine	0.41	0.23	0.60	1.3E‐03	777
C40:6 phosphatidylcholine	−0.33	−0.50	−0.16	7.9E‐03	1012
Theophylline	0.09	0.04	0.14	2.1E‐02	1007

^a^ Number of participants following exclusions of undetectable levels or levels outside of 6 SDs.

Finally, the associations of aspartame or saccharin intakes with plasma metabolites were examined among those with a BMI < 25 kg/m^2^. Most previous associations were rendered non‐significant compared to the main analyses; however, magnitudes of association remained similar. Heavy aspartame intake remained strongly associated with saccharin (*β* = 1.06; *q* = 3.0E‐22) and nominally related to 5‐acetylamino‐6‐amino‐3‐methyluracil (*β* = 0.34; *q* = 0.15), 1‐methylurate (*β* = 0.34; *q* = 0.15), and 1,7‐dimethyluric acid (*β* = 0.32; *q* = 0.20). Saccharin intake remained related to plasma levels of saccharin alone (*β* = 0.67; *q* = 1.3E‐08).

## 4 Discussion

This is the first metabolomics study to test whether individual intakes of aspartame or saccharin are associated with plasma metabolites that may suggest disruptions in metabolic homeostasis; we further aimed to control for the reverse causation bias present in previous studies. While we detected associations of caffeine metabolites and myo‐inositol—which likely reflect the ArtSws in beverages (aspartame and inositol)—we found no evidence that either aspartame or saccharin was associated with plasma metabolites indicative of metabolic disruptions. Multiple sensitivity analyses and PSM confirmed these findings.

### 4.1 Null Associations Suggest no Detectable Metabolic Disruptions With ArtSw Intake

The core finding of the present analysis was the absence of associations of aspartame or saccharin intake with hundreds of plasma metabolites—consistent with a null effect of either ArtSw on metabolic function. Had the alternative hypothesis been upheld, numerous plasma metabolite signals would have been detected. Among these, plasma levels of the branched‐chain amino acids (valine, isoleucine, and leucine) [19], glutamate [20], glucose, and lactate [21] as well as multiple lipid classes including the glycerophosphates, triglycerides, ceramides, sphingolipids, or acylcarnitines [22] have been previously related to, or otherwise shown to predict, insulin resistance or incidence of adverse metabolic health outcomes. Yet, no signals for these or other metabolites indicating metabolic dysregulation were detected in the primary analysis, which is inconsistent with ArtSw inducing metabolic dysfunction.

Results for subsequent PSM and sensitivity analyses were largerly in agreement; though two additional and relatively strong associations in terms of effect sizes were detected with aspartame that must be acknowledged. Following the exclusion of non‐aspartame users, aspartame (per SD) was found to be inversely associated with C40:6 phosphatidylcholine (*β* = −0.34; *q* = 3.1E‐03). This observation is indeed consistent with aberrant lipid metabolism, and plasma levels of C40:6 phosphatidylcholine have been inversely associated with insulin resistance as determined by HOMA‐IR in older adults [23]. However, in our analyses, we found no supporting evidence from other phosphatidylcholine species, precursors, or metabolites of C40:6 metabolism (arachidonic acid, docosahexaenoic acid, or choline)—which were also tested. Apart from this signal, the PSM analysis showed a reasonably strong association of heavy aspartame intake with plasma glucuronate (per SD) (*β* = 0.30; *q* = 3.4E‐02), a metabolite of myo‐inositol [24], which is not suggestive of metabolic perturbations.

The absence of the association between saccharin intake and plasma saccharin levels when analyzed continuously and restricted to consumers is unclear. This attenuation may reflect limited statistical power (*N* = 219), inter‐individual variability in saccharin absorption or clearance, imprecision in dietary intake estimation, or a combination of these factors.

### 4.2 Previous Findings

To date, no previous cohort study has aimed to identify associations between individual ArtSws and plasma metabolites, but a metabolomics study of diet soda intake was recently conducted in an Atherosclerosis Risk in Communities Study (ARIC) subcohort [25]. Despite the > 20‐year gap between dietary intake collection (1987–1989) and the plasma collection that was used for metabolomics analysis (2010 and 2014), 11 of 360 metabolites were found to be associated with diet soda intake following correction for multiple comparisons. While a modest signal was detected for greater plasma glucose (*β* = 0.12; 95% CI: 0.09, 0.16) in heavy diet soda consumers (≥ 1 serving/day), the remaining 10 associations were not suggestive of metabolic dysfunction. Indeed, the strongest associations were observed for diet soda additives including mannitol, gluconate, erythronate (erythritol metabolite), threonate (ascorbic acid metabolite), and caffeine. The investigators posited that associations with metabolites like tryptophan, betaine, and N6‐acetyllysine likely reflected healthier dietary choices among those who consume diet sodas. This conclusion is consistent with the modest but significantly greater HEI2020 scores among consumers of aspartame and saccharin intake found in CARDIA participants (Tables 1 and 2). Most of the significant metabolites reported in ARIC were analyzed in the present analysis, but were not significantly related to aspartame or saccharin intake.

### 4.3 Ongoing Controversy and Implications for Future Research

There remain several core controversies and hypothetical mechanisms involving ArtSws and their putative effects on metabolic health. Specifically, ArtSw intake has been shown to induce acute salivary and insulin secretions [26], which have been proposed to affect appetite and caloric intake [27] as well as the cephalic phase insulin release [28]. And yet, we found no corroborating evidence that these acute phenomena have detectable consequences in the plasma metabolome. Moreover, in addition to their null associations with most plasma metabolites, aspartame and saccharin consumers showed lower caloric intakes, which is inconsistent with any influence on appetite.

Microbiome alterations and dysbiosis that promote glucose dysregulation remain a central issue in the ArtSw debate, and mixed results in trials and observational studies have been reported so far [29, 30, 31]. From a physiological standpoint, a *direct* effect of aspartame or saccharin on the human microbiome is unlikely. Aspartame has been shown to be metabolized by gastrointestinal enzymes into its phenylalanine, aspartic acid, and methanol components [32], which are then absorbed [33]. By contrast, dietary saccharin is not metabolized in the gastrointestinal system, and up to 95% is absorbed in its native form and excreted in urine [32, 34]. Therefore, these two ArtSws are largely *not* in direct contact with the gut microbiome. However, it is well established that *the microbiome is modified by* the *nutrient composition of dietary intake* [35, 36, 37], and differences in diets and diet quality among ArtSw consumers versus non‐consumers may be a critical variable in real‐world settings [38, 39]. Further research is needed to more thoroughly interrogate the potential role of diet quality in ArtSw consumers and non‐consumers.

### 4.4 Strengths and Limitations

This study has a number of strengths. First, the primary analysis included over 2000 Black and White CARDIA participants from four centers across the United States. This provided both external validity and the largest metabolomics study sample to test these individual dietary exposures. Additionally, the large sample size in CARDIA allowed for PSM and multiple sensitivity analyses. While PSM reduces the sample size, it also reduces the probability of confounding inherent in observational studies, and the results largely supported the robustness of the main findings. In terms of data collection, standardized CARDIA dietary assessment queried brand name information, and this allowed for the estimation of individual ArtSw intakes. Moreover, the depth of dietary collection allowed us to generate scores for healthy eating and control for its potential confounding, coupled with total caloric intake. Finally, we measured 549 unique plasma metabolites, and this allowed us to scrutinize hundreds of potential pathways, including those related to amino acid, carbohydrate, and lipid metabolism.

Several limitations must also be acknowledged. First, this was a cross‐sectional analysis, and causation is difficult to infer. While this limitation is mitigated by the improbability of reverse causation, that is, it is biologically implausible that circulating metabolites drive consumption of aspartame or saccharin—longitudinal studies and randomized trials focused on metabolomic endpoints are needed to strengthen causal interpretations. Second, despite adjustment for demographic, behavioral, and clinical covariates, residual confounding by unmeasured factors cannot be ruled out. To reduce this possibility, we conducted multiple sensitivity analyses and propensity score matching, which yielded consistent results. Third, statistical power in the primary analyses was limited by categorizing aspartame and saccharin intakes, which was necessary due to the large numbers of non‐consumers. However, follow‐up sensitivity analyses of aspartame and saccharin as continuous variables, as well as the PSM analysis, were well‐powered and confirmed the strongest findings of the primary analyses. Fourth, we assessed approximately 540 unique metabolites using targeted metabolomics approaches. Although unlikely, we cannot rule out the possibility that aspartame or saccharin are associated with unmeasured metabolites that, in turn, affect aspects of metabolic homeostasis that are not captured by our measurements. Fifth, the CARDIA cohort is composed of Black and White participants recruited from four field centers; however, the generalizability of our findings to other races, ethnicities, geographic areas, and metabolic health remains a limitation. Finally, we did not have the statistical power to test associations of extreme ArtSw intakes, and we were therefore unable to evaluate whether they may be related to metabolic perturbations.

## 5 Conclusions

In what is the largest metabolomics study of ArtSws, we provide novel evidence that real‐world intakes of aspartame and saccharin are not associated with plasma metabolites independent of other factors including adiposity, total caloric intake, and diet quality. These findings do not support aspartame or saccharin intake as a risk factor for metabolic dysfunction; however, they should not be generalized to more extreme intakes of these ArtSws.

## Author Contributions

B.T.S. conducted the data analysis, interpreted the results, and composed the first draft of the manuscript; D.R.J. contributed to the interpretation of the data, reviewed and edited the manuscript for content. E.R.L., C.C., V.L.M., L.V.H., J.G.T., and J.J.C. critically reviewed the manuscript content and provided important revisions. L.M.S. contributed to the design, acquisition, and interpretation of the data, and reviewed/revised the manuscript for important content. All authors approved the submitted manuscript.

## Conflict of Interest

V. Murthy owns stock in General Electric, Cardinal Health, Viatris, Pfizer, Amgen, Merck, and Johnson & Johnson and stock options in Ionetix. He is a paid consultant for INVIA Medical Imaging Solutions & Siemens Healthineers. V. Murthy has received research support through his institution from Siemens Healthineers. The remaining authors declare no conflict of interest.
